# An Analysis of China’s Onshore and Offshore Exchange Rates—Adjusted Thermal Optimal Path Approach Based on Pruning and Path Segmentation

**DOI:** 10.3390/e21050499

**Published:** 2019-05-15

**Authors:** Dawen Yan, Kin Keung Lai

**Affiliations:** 1 School of Mathematical Sciences and Faculty of Management and Economics, Dalian University of Technology, Dalian 116024, China; 2 College of Economics, Shenzhen University, Shenzhen 518060, China

**Keywords:** offshore CNH spot market, onshore CNY spot market, local lead-lag relationship, adjusted thermal optimal path method, daily closing price change, bid-ask spread

## Abstract

The study of the lead-lag relationship between the Hong Kong offshore Renminbi (CNH) spot market and onshore (CNY) spot market is of great importance for its wide application in market risk management. In this paper, we study the correlation between the CNH and CNY spot markets in the contexts of daily closing price change and the 2011–2016 Bid-Ask spread (BAS). We test the existence of causality relation between CNH/CNY pairwise change and BAS by using the conventional method of vector auto-regression (VAR) model in the observation period. Furthermore, we detect the local lead-lag dependence relationships between CNH/CNY pairwise change and BAS by using a non-parametric approach-adjusted Thermal Optimal Path (TOP) method. Through introducing a Pruning and Path segmentation algorithm, we address the problem of computation infeasibility that may be encountered in application of the existing TOP method for the detection of lead-lag relationship between two time series with long time duration. Theoretical analyses and simulation results are presented to verify validity of adjusted TOP method in the setting of big time-series data set. This study also provides some interesting findings: (1) the offshore CNH market is informationally integrated with the onshore CNY market from two aspects of closing price change over two consecutive single days and BAS used as a proxy for market liquidity; (2) Local dependency between the two markets changes with economic conditions changing, which would facilitate both investor and policy maker decision making.

## 1. Introduction

The offshore CNH market was created in the early 2000s for meeting the needs of Chinese Renminbi (RMB) deposits, currency convertibility and cross-border consumption of Hong Kong residents at that time. Owing to a series of measures of Chinese government, the offshore CNH market has grown rapidly and played an increasingly important role in RMB liberalization. In recent years, daily RMB offshore spot trading volume has expanded from a negligible amount to $1.3 billion daily, while bulk of trades are conducted in Hong Kong given its proximity and shared time zone with the Chinese mainland as well as its deeper liquidity [1].

Compared with the onshore CNY market, since Hong Kong is a world financial and trade center, CNH is more inclined to be influenced by international economic and financial conditions and reflect the supply of and demand for RMB in the international market. The CNY market is relatively closely linked with the Chinese government policies and may not fluctuate significantly due to the regulation of the People’s Bank of China (PBC). Although obvious differences between CNY market and CNH market exist, correlation between these two markets, especially price changes, increases significantly with the offshore CNH market’s development over almost twenty years [2].

Accurately detecting the relationship between CNY and CNH market has become increasingly important for both investors involved in the foreign exchange market (FX) and policy makers. Therefore, CNH/CNY cross-market price discovery and information spillover has drawn much attention from many researchers in recent years. Wu and Pei point out that the price of CNY guides the price of CNH, while the price of non-deliverable forward (NDF) affects those of CNH and CNY [3]. Leung and Fu find that the spillover effect of CNH and CNY is bidirectional. However, the effect of CNY on CNH is larger. They believe that CNY will continue to play a leading role in the future [4]. Cheung and Rime also exhibit the short-term interactions between CNH return and CNY return by using the vector auto-regression correction model (VECM). Their analyses show that short-term interaction between CNH spot market and CNY spot market changes with time changing, but the influence of CNH spot on CNY spot is increasingly stronger towards to the end of sample period in general [2]. Du and Lai employ the time-invariant Student-t copula method to capture the co-movement between offshore spot rate and the onshore spot rate [5]. Shi finds that CNH market, with high information advantage and price discovery function, has become the dominant market [6].

The literatures above indicate that CNH offshore market has been informationally integrated with CNY onshore market. CNY market may dominate CNH market, but the influence of CNH on CNY is increasingly significant, with the offshore CNH market’s development over almost twenty years. Proceeding along this line, this paper detects lead-lag dependency relationship between CNH and CNY spot market from two aspects of price change and liquidity based on parametric method of vector auto-regression model and non-parametric method of adjusted Thermal Optimal Path approach.

The “Thermal Optimal Path” (TOP) method or the “Optimal Thermal Causal Path” method, as a non-parametric statistical method was proposed by Sornette and Zhou [7]. It is viewed as an extension of time distance measurements and can be used to reveal a prior arbitrary non-linear dependence structure of lead-lag between two time series in different periods. This method, which reflects sufficiently the structure and character of data, has been broadly used to many economic and financial problems, such as the study of the relationship between inflation and economic growth rate [8,9], the study of the relationship between credit and financial crisis [10], the study of the relationship between stock index and GDP [11], stock price and investor sentiment [12] and stock index and stock index futures [13], the study of links between UK and US real-estate and monetary policies [14] and the study of the lead-lag relationship between the spot and futures markets [15] and different countries’ future markets [16] etc. We also find that some researchers have applied TOP method to the relevant study with Chinese exchange market. For example, Wang et al. employ the TOP method to explore the long-run dynamic relationship between CNY/USD exchange rate and the corresponding attention index. They find that the significant inter-dependency exists and the change of exchange rate is 1–2 days lag behind the attention index [17]. Xu et al. apply the TOP method to detect the interaction patterns between the onshore CNY and offshore CNH exchange rates [18]. 

Our study is closely related to the paper of Xu et al. [18], but there are two obvious differences between their work and ours. First, we propose an adjusted TOP method which combines Path Segmentation and Pruning methods mainly to solve the computational infeasibility of TOP method under the situation of CNH/CNY pair big data, while Xu et al. [18] consider the interaction between CNH/CNY spot rate through using the existing TOP method. Second, the focus is different. We discuss the lead-lag dependency relationship between CNH and CNY spot market from two aspects of price change and liquidity, while Xu et al. [18] focus on the detection of interaction between the CNH and CNY closing price. 

In practical application of TOP method, we find that the size of the used time series data and data value can influence its implementation, and there may be computational infeasibility in the case of medium or large size sample. Such problem mainly results from that the cumulative Boltzman factors used to obtain the optimal thermal path tend to be unacceptably large with size of used data increasing. In the existing related studies, the researchers try to avoid such problem of computational infeasibility either by using low frequency data to reduce the number of used time series data [7], or by eliminating the numerous and possible starting and ending points in order to reduce the computational cost [13]. However, these two ways seem not always to be suitable and effective, because high frequency data such as daily data, minute-scale data even second-scale data may need to be used and considered in many practical cases, and size of used data will generally continue to increase in the future. Moreover, once the size of data sample becomes relatively large, the cumulative Boltzman factor tends to be infinity with number of recursion. Therefore, the problem of computational infeasibility of TOP method cannot be solved by only eliminating numerous starting and ending points. In order to fully address the problem, we propose an adjusted TOP method which can eliminate the effect of size of data sample and data value and enhance the popularity of TOP method to some extent. 

Another focus of this paper is to detect the dependence relationship between CNH and CNY market liquidity. Foreign exchange market liquidity, as a significantly important indicator to measure market’s efficiency and resilience describes the degree to which the foreign currency transactions, especially large transactions can be quickly executed with a small impact on prices [19], thus gaining widespread concern from all market participants including investors and central banks of all countries. Compared with CNY market liquidity, the liquidity in CNH market is relatively lower [20]. In the early days of the offshore RMB development, CNH market liquidity has been low due to CNH immaturity and expectations of appreciation from investors. Investors tend to hoard CNH in deposit accounts and expect higher return in the first instance. After 2010, CNH market has moved into a new phase, but the liquidity in CNH market seems to be at a limited level. This may be caused by the strict restrictions on capital flows from people’s bank of China (PBC) and the Hong Kong monetary authority (HKMA). The low liquidity may thwart RMB internationalization and reduce associated business with CNH in the financial hub of Hong Kong as a consequence [21]. Therefore, discussions on RMB markets liquidity have been mostly around how to promote CNH liquidity (e.g., Cushnie [20], Eraslan [21], Craig [22], Funke [23], Danese [24] and Hui [25]). To the best of our knowledge, there are little researches on the quantitative analysis of correlation between CNY liquidity and CNH liquidity. This study fills the gap in the literature. 

The remaining parts of this paper are organized as follows: Section 2 proposes the adjusted Thermal Optimal Path method and applies it to some simple numerical example to compare its performance with the existing TOP method. Section 3 presents an application of the adjusted Thermal Optimal Path on dependence relationship between CNH and CNY spot market. We detect the lead-lag dependency of CNH/CNY spot from two aspects of closing price change over two consecutive single days and Bid-Ask spread (BAS) used as a proxy for market liquidity. Besides, causality analysis based on vector auto-regression model for the first difference series of CNY/CNH daily closing price and CNY/CNH BAS series are respectively implemented, which may be regarded as a preliminary analysis of relationship between CNH and CNY spot before conducting TOP method. All empirical results and corresponding analyses will be presented in this section. Section 4 concludes.

## 2. Methodology 

### 2.1. Thermal Optimal Path Method

The thermal optimal path (TOP) method, also called thermal optimal causality path method can be viewed as complementing the Granger causality test method [7]. This method can detect the non-linear local dependence structure of time-series and reflect the degree and character of dependence in the different periods of time, which provides a new research perspective of the relationship between the two time series. The details about TOP method are as follows.

Consider two time series of {*X*(*t*_1_), *t*_1_ = 0,1,2,…,*n*} and {*Y*(*t*_2_), *t*_2_ = 0,1,2,…,*n*}. Suppose, here *X*(*t*_1_) and *Y*(*t*_2_) represent CNH and CNY spot series respectively. Through absolute distance, we define the distance between the realization of the first time series at *i*-*th* time point *t*_1,*i*_ and the realization of the second time series at *j*-th time point *t*_2,*j*_:

*ε*(*t*_1,*i*_,*t*_2,*j*_) = |*X*(*t*_1,*i*_) − *Y*(*t*_2,*j*_)|, (1)

where ε(*t*_1__,*i*_, *t*_2*j*_) is called local distance or local energy. 

The main idea behind TOP method is to construct a mapping Φ from times {*t*_1_} of the first time series *X*(*t*_1_) and the times {*t*_2_} of the second time series *Y*(*t*_2_) that ensures summation of local distance between *X*(*t*_1_) and *Y*(*t*_2_) minimal, i.e.:

(2) $$\Phi \left(t_{1}\right)=\left\{t_{1}\right\}\to \left\{t_{2}\right\}=\{\Phi \left(t_{1,i}\right):\;\;min\overset{n}{\underset{i=1}{\sum }}\varepsilon \left(t_{1,i},\Phi \left(t_{1,i}\right)\right)\}$$

Equation (2) implies that the correlation between *X*(*t*_1_) and *Y*(Φ(*t*_1_)) is closest in the sense of minimal difference in values of these two time series, i.e., *X*(*t*_1_) and *Y*(Φ(*t*_1_)) match best. Furthermore, the following constraint is added to ensure monotonicity and continuity of mapping function Φ:

(3) $$0\leq \Phi \left(t_{1,i+1}\right)-\Phi \left(t_{1,i}\right)\leq 1.$$

Let *E*(*t*_1,*i*_,*t*_2,*j*_)denote the summation of absolute distance of the optimal path starting from some original point of (*t*_1_,*t*_2_) and ending at some specific point (*t*_1,*i*_,*t*_2,*j*_), i.e., the cumulative absolute distance between *X*(*t*_1_) and *Y*(Φ(*t*_1_)) or minimum energy from the predetermined starting point of time to predetermined ending point of time. Under constraint (3), *E*(*t*_1_,*t*_2_) follows the fundamental relation in (4),

*E*(*t*_1,*i*_,*t*_2,*j*_) = *ε*(*t*_1,*i*_,*t*_2,*j*_) + *Min*[*E*(*t*_1,*i*_ − 1, *t*_2,*j*_),*E*(*t*_1,*i*_, *t*_2,*j*_ − 1),*E*(*t*_1,*i*_ − 1, *t*_2,*j*_ − 1)]. (4)

The key insight in (4) captured by this equation is that the minimum energy path that reaches point (*t*_1,*i*_,*t*_2,*j*_) can only come from one of the three points (*t*_1,*i*_−1,*t*_2,*j*_−1), (*t*_1,*i*_−1,*t*_2,*j*_) and (*t*_1,*i*_,*t*_2,*j*_−1) preceding it. Then, the minimum distance path reaching (*t*_1_,*t*_2_) is nothing but an extension of the minimum energy path reaching one of these three preceding points, determined from the minimization condition (3). Thus, the global minimization procedure is fully determined once the starting and ending points of the paths are defined. Since the lead-lag between the two time series can be anything at any time, we allow the starting point to lie anywhere on the horizontal axis *t*_2_ = 0 or on the vertical axis *t*_1_ = 0. Similarly, we allow the ending point to lie anywhere on the horizontal axis *t*_2_ = *n* or on the vertical axis *t*_1_ = *n*. This allows for the fact that one of the two time series may precede the other. For each given pair of starting and ending points, we obtain a minimum energy path. The minimum energy path over all possible starting and ending points is then the solution of our global optimization problem (2) under constraint (3). This equation of this global optimal path defines the mapping *t*_1_ and *t*_2_ defining the dependence relationship between the two time series.

An optimal path can be obtained by the dynamic optimization method shown as above, but the path may reflect a spurious relationship between {*X*(*t*_1_)} and {*Y*(*t*_2_)} due to the noise. Therefore, a thermal optimal path method is applied to reduce the interference of noise. The main procedures to obtain thermal optimal path are as follows:

Step 1: for the convenience of analysis, we use the following transformation of axes *t*_1_ and *t*_2_:

(5) $$\{\begin{array}{c} x=t_{2}-t_{1}\\ t=t_{2}+t_{1} \end{array},$$

where *t* and *x* are interpreted as an effective time and the position of a path at time *t* respectively. The variable *x* directly quantifies the lead-lag relation between the two series by definition, and a positive (negative) *x* means that the first time series {*X*(*t*_1_)} leads (lags) the second time series {*Y*(*t*_2_)}. 

Step 2: add Boltzman weight factor for reduction of noise. We define:

(6) G⊲(xi,j,ti,j) =∑ce−EcT.

In expression (6), *c* is one path starting from (0,0) and ending at (*t_ij_*, *x_ij_*) where *t_ij_* = *t*_2,*j*_ + *t*_1,*i*_ and *x_ij_* = *t*_2,*j*_ − *t*_1,*i*_. In (6) *E_c_* is defined as the cumulative energy, i.e., cumulative absolute distance in the given path *c*; *T* is the “temperature” for the path *c*; e−EcT is defined as Boltzman factor corresponding to path *c*; $G_{⊲}\left(x_{i,j},t_{i,j}\right)$ is the sum of Boltzmann factors over all path starting from (0,0) and ending at (*t_ij_*, *x_ij_*). In the definition of (6), Boltzmann factor corresponding to one path decreases with the energy in this path increasing, which implies that the weight for the path with higher accumulated energy above the minimum energy in optimal path is smaller. Obviously, the weight for absolute minimum energy path obtained by solving the global optimization (2) under constraint (3) is larger than any other path. Temperature *T* is a key parameter and quantifies how much deviation from minimum energy is allowed. In brief, the smaller *T* is, the larger the probability that the optimal path with minimum energy is only used ignoring interference of noise is; while conversely, the larger *T* is, the larger the probability with that other path are used to average out noise contribution is. In the subsection, we show the sensitivity analysis of *T*.

Step 3: compute the Thermal optimal path and its variance. We use the following formulas:

(7) $$\langle x(t)\rangle =\sum_{x}x\left(t\right)\;G_{⊲}\left(x,t\right)/G_{⊲}\left(t\right),$$

(8) $$\sigma_{x}^{2}=\sum_{x}{\left(x\left(t\right)\;-\langle x\left(t\right)\rangle \right)}^{2}G_{⊲}\left(x,t\right)/G_{⊲}\left(t\right),$$

(9) $$G_{⊲}\left(t\right)=\sum_{x}G_{⊲}\left(x,t\right),$$

where 〈x(t)〉 defined as average position at *t* is the thermal optimal causality path between time series *X*(*t*_1_) and *Y*(*t*_2_); $\sigma_{x}^{2}$ defined as variance assesses the uncertainty of deviation from the optimal thermal path. The optimal thermal path 〈x(t)〉 takes into account the set of neighboring (in energy) paths, which allows one to average out the noise contribution to the distance matrix *E*(*t*_1,*i*_,*t*_2,*j*_). 〈x(t)〉 reflects the degree of time lag or time lead between *X*(*t*_1_) and *Y*(*t*_2_) for different periods of time. Positive (negative) 〈x(t)〉 indicates that the time of *Y*(*t*_2_) is greater (less) than the time of *X*(*t*_1_) when time series *X*(*t*_1_) and *Y*(*t*_2_) match best in the sense of expression (2). This implies that *X*(*t*_1_) leads (follows) *Y*(*t*_2_). 〈x(t)〉 is main result we show in the next section. 

### 2.2. Improvement of TOP Method 

The existing thermal optimal path (TOP) method suffers from two main computational limitations, although it is a comprehensible and conceptually appealing approach for detecting lead-lag dependency relationship between two time series. The *first limitation* lies in the fact that the calculations of the Boltzman factor weights at some time points cannot be implemented based on the existing TOP method, and thus Equation (7) may provide an inaccurate average thermal optimal path 〈x(t)〉. The reasoning is that the calculations of some of Boltzman factor weights may involve calculation of cumulative distance of those paths emanating from origin (0,0) ending at a point beyond the period of observation, resulting from the expanded range of time variable *t* = *t*_1_+ *t*_2_ after coordinate translation is from (*t*_1_,*t*_2_) to (*x*, *t*). On this limitation of the existing TOP method, we provide more detailed descriptions below. 

Let us recall a formula of 〈x(t)〉 presented in the paper of Sornette and Zhou [7] and shown by:

(10) $$\langle x\left(t\right)\rangle =\sum_{x=-t:2:t}xG_{⊲}\left(x,t\right)/G_{⊲}\left(t\right).$$

Equation (10) can be equivalently transformed to the following equation after using the coordinate’s transformation from (*x*, *t*) to (*t*_1_, *t*_2_) shown in (5):

(11) $$<x(t)>=\sum_{i=0}^{t}\left(t-2i)g(i,t-i\right)/\sum_{i=0}^{t}g(i,t-i)\;,\;t=\;0,1,\text{…},2n.$$

Here term *t −* 2*i* represents the time lag for a given *t*; *g*(*i,t − i*) represents the sum of Boltzmann factors, i.e., cumulative distance over all paths starting from (0,0) and ending at (*i,t − i*) in coordinate system (*t*_1_*,t*_2_); *n* + 1 represents number of data, thus *n* represents maximum subscription of sample. Equation (11) is actually used to determine the average thermal optimal path 〈x(t)〉. However, it is not difficult to see that there are no definitions for some *g*(*i,t − i*) when either *i* or *t − i* is greater than *n*. This is because that *g*(*i,t − i*) is determined by sum of the local distance *ε*(*t*_1,*i*_,*t*_2,*j*_) where *ε*(*t*_1,*i*_,*t*_2,*j*_)is defined when *i* and *j* are between 0 and *n,* otherwise there is not definition for *ε*(*t*_1,*i*_,*t*_2,*j*_). For example, *ε*(0,2*n*) is not defined, because *ε*(0,2*n*) is defined by |*X*(*t*_0_) *− Y*(*t*_2*n*_)| based on (1), where *Y*(*t*_2*n*_) is not included in the observed sample set {*Y*(*t*_0_),*Y*(*t*_1_),…,*Y*(*t_n_*)}. However, calculation of 〈x(2n)〉 needs to use value of *g*(0,2*n*), in other word, the value of *ε*(0, 2*n*).

Aiming at addressing this limitation, this paper provides a Pruning Algorithm which can be considered to improve accuracy of TOP method. A natural way to solve the calculation problem of Boltzman factor discussed above is to just eliminate those weights of *g*(*i,t* − *i*) without definition. Therefore, besides using formula (11) for obtaining 〈x(t)〉, we add the following constraints:

(12) $$\{\begin{array}{c} 0\leq i\leq n\\ 0\leq t-i\leq n \end{array}\Leftrightarrow \{\begin{array}{c} 0\leq i\leq n\\ t-n\leq i\leq t \end{array}\Leftrightarrow \max\left\{0,t-n\right\}\leq i\leq \min\left\{n,t\right\}.$$

Replacing lower bound 0 and upper bound *t* with max{0,t−n} and min{n,t} respectively, we have:

(13) $$<x(t)>=\sum_{i=\max\{0,t-n\}}^{\min\{n,t\}}\;(t-2i)g(i,t-i)/\sum_{i=\max\{0,t-n\}}^{\min\{n,t\}}\;g(i,t-i),\;t=0,1,\text{…},2.$$

In Equation (13), all *g*(*i,t − i*) can be obtained based on the previous definition about cumulative distance, so value of 〈x(t)〉 for any *t* between 0 and 2*n* can be obtained. Those *g*(*i,t − i*) in that *i* does not satisfy condition (12) are eliminated from formula 〈x(t)〉. Equation (13) presents the result of TOP method after the first Pruning. 

The key idea of the Pruning Algorithm lies in directly eliminating those almost impossible choices or paths in the process of obtaining average thermal optimal path. For example, when either *i* or *t − i* is greater than *n*, any path starting from (0,0) ending at (*i,t − i*) in the case of size of sample equal to *n* can be regarded as an impossible path. After the first Pruning, or adding constraint of *i* shown in (12), it is not difficult to find that absolute value of lag term *t −* 2*i* is not great than *n*. In other words, the maximum lag quantity cannot exceed *n* that can be regarded as the length of period of observation. However, further pruning, i.e., further eliminating impossible paths needs to be considered to improve TOP method performance and reduce computation and memory requirement. 

Besides (12), the condition |*t* − 2*i*| ≤ *β* (0 *≤ β ≤ n* and *β* is predetermined nonnegative integer) is added to control lag term within an acceptable range from a practical point of view. Adding |*t* − 2*i*| ≤ *β* (*i* is nonnegative integer) to the equivalent expressions (12), then we have:

(14) $$\{\begin{array}{c} \left|t-2i\right|\leq \beta \\ 0\leq i\leq n\\ 0\leq t-i\leq n \end{array}\Leftrightarrow \{\begin{array}{c} \frac{t-\beta }{2}\leq i\leq \frac{t+\beta }{2}\\ 0\leq i\leq n\\ t-n\leq i\leq t \end{array}\Leftrightarrow \;\max\left\{0,t-n,\frac{t-\beta -\min\{0,\;{\left(-1\right)}^{t+\beta }\}}{2}\right\}\leq i\leq \min\left\{n,t,\frac{t+\beta +\min\left\{0,\;{\left(-1\right)}^{t+\beta }\right\}}{2}\right\}.$$

Let *δ* = min{0,(−1)*^t^*^+*β*^}. *δ* is 0, if both *t* and *β* are even numbers or odd numbers; otherwise, *δ* is −1. Under condition (14), Equation (13) can be further transformed to:

(15) $$<x(t)>=\sum_{i=\max\{0,t-n,\frac{t-\beta -\delta }{2}\}}^{\min\{n,t,\frac{t+\beta +\delta }{2}\}}\;(t-2i)g(i,t-i)/\sum_{i=\max\{0,t-n,\frac{t-\beta -\delta }{2}\}}^{\min\{n,t,\frac{t+\beta +\delta }{2}\}}\;\;g(i,t-i),t=\;0,1,\text{…},2n.$$

Obviously, when *β = n*, conditions (14) and (12) are equivalent, and (15) and (13) are equivalent. (15) under constraint (14) is used to determine 〈x(t)〉 which implies that lag term exceeding this range, i.e., the ones greater than *β* (or *β −* 1) or less than −*β* (or −*β* + 1) are directly eliminated in the process of obtaining 〈x(t)〉. More specifically, the value of lag term (*t*−2*i*) changes from *β* to −*β* when both *t* and *β* are odd numbers or even numbers, otherwise the value of lag term (*t − * 2*i*) changes from *β −* 1 to −*β* + 1. This can be regarded as further path pruning. It should be the case. For example, in this paper, we detect local lead-lag dependency relationship between CNH and CNY spot exchange rate. If lead-lag relationship exists, it means that CNH spot guides or lag CNY spot, i.e., changes in closing price of CNH spot rate happened before or after similar changes happened in CNY spot market. However, even though the lead-lag relationship exists; the possible time lag should fall within a certain range. Such event that current CNY(CNH) spot rates will repeat the change happened in CNH(CNY) spot market a half of year ago or a year ago may seldom occur. In this paper, we assume that *β* = 30 (one month), i.e., the maximum lag does not exceed one month within the period of observation. The influence of different valves of *β*, e.g., 3 (days), 7 (one week) and 15 (half of a month) on the results of thermal optimal path will be present in Section 3. 

More paths are eliminated through further pruning. It seems to provide computational shortcuts. Even those accumulative Boltzman factors *g*(*i,j*) that don’t satisfy condition (14) are not needed to compute for determining 〈x(t)〉 after Pruning. We are still confronted with another problem when we implement TOP method to detect lead-lag dependency relationship between CNH and CNY spot. That is the values of lots of *g*(*i*,*j*) are unacceptably large which results in failure to obtain 〈x(t)〉. For example, for determining 〈x(1250)〉, *g*(620,630) is needed to be known and used, but the value of *g*(620,630) displayed in computer is “infinity”. This involves the second limitation of use of TOP method. 

The *second limitation* lies in that big sample size may result in computational infeasibility of the TOP method. The computational quantity and time grows rapidly as number of data, *n*. By either using formula (4) to obtain minimum energy path or using (11) to obtain the average thermal optimal path, ones can be confront with the problem of computational infeasibility in the case of medium or large size sample. In our case, the number of time series data during the observation period is 1250 (see the following Section 3).This implies that numbers of possible starting points and ending points are 2*n* − 1 = 2 × 1250 − 1 = 2499 and 2*n* − 1 = 2 × 1250 − 1 = 2499 respectively, based on the preceding analyses. Thus, there are millions (2499^2^) minimum energy paths between each possible starting point and ending point. It must involve computation and comparison of cumulative energy of millions even ten millions possible paths, in order to obtain the global optimal thermal path by using recursive Equation (4).

On the other hand, determination of average thermal optimal path by using Equation (7) or (11) may incur computational infeasibility in the case of big sample. *g*(*i*,*j*) may significantly increases as *i* and *j* increase because of the form of definition of *g*(*i*,*j*). In our case, the empirical results show that the maximum value of *g*(*i*,*j*) has already reached 4.2888 × 10^180^ for *i*,*j* between 250 and 500. For *i*,*j* greater than 510, the value of *g*(*i*,*j*) displayed in computer is “infinity”. Unacceptably large valves of *g*(*i*,*j*) result in computational infeasibility of average thermal path. In fact, eliminating the numerous and possible starting and ending points or Pruning Algorithm mentioned above cannot solve the problem when the time series data size is relatively big. 

To resolve this limitation, this paper proposes the Path Segmentation method. We combine Path Segmentation and Pruning methods to solve the computational infeasibility of the TOP method in big sample size situations. Path Segmentation is mainly used to determine 〈x(t)〉 when *t* is relatively large. When *t* is big, all *g*(*i*,*t* − *i*) tend to take unacceptably large values in Equation (15). We try to divide the path starting from origin (0,0) ending at (*i*,*t − i*) into two pieces: the one from origin to a point on the path; the another one from the ending point of previous sub path to (*i*,*t − i*).Through path segmentation, *g*(*i*,*t − i*) can roughly be described the multiplication of two cumulative Boltzman factors corresponding two sub paths. The Boltzman factor corresponding the first sub path as a common factor will be canceled from all *g*(*i*,*t − i*) terms in the numerator and denominator in formula (15). The cumulative Boltzman factors from a new chosen point to (*i*,*t − i*)are practically used to determine 〈x(t)〉 but the complete cumulative Boltzman factors from (0,0) to (*i*,*t − i*) when *t* takes relatively big value. The purpose of this lies in that calculation and use of some cumulative distance will be ended before its value reaches to “infinity” or unacceptably large. This can avoid computational infeasibility resulting from unacceptably large weight of sum of Boltzman factor *g*(*i*, *t − i*). We present the complete Path Segmentation method in the Appendix, because the whole analytical process is a little bit long, which would make reading inconvenient. Interested readers may check more details there. We only show the main procedures of Adjusted TOP method proposed by this paper in Algorithm 1. 

**Algorithm 1.** Adjusted TOP method based on pruning and path segmentation algorithm
**Pruning and Path Segmentation algorithm**

Construct local distance matrix *ε*(*t*_1,*i*_,*t*_2,*j*_), for *i, j* = 0,1,2,…,*n*;Use formula (15) to determine the average thermal optimal path 〈x(t)〉, when the maximum value of independent variable *i* of *g*(*i*,*t −* *i*) used in (15) to compute 〈x(t)〉 is less than n*. Use formula (A12) in Appendix to determine the average thermal optimal path 〈x(t)〉,when the maximum value of independent variable *i* of *g*(*i*,*t* − *i*) used in (15) to compute x(t) is between n* and n* + *n*_1_ − *β*.Use formula (A14) in Appendix to determine the average thermal optimal path x(t),when the maximum value of independent variable *i* of *g*(*i*,*t − i*) used in (15) to compute x(t) is between n* + *n*_1_ − *β* and *n*.
Note: the concrete definition and description of n*, *n*_1_ in Algorithm 1 can be funded in Appendix. In the following empirical study, *n* = 1249 (time-series data size) and we set *n*_1_ = 420 (a partition coefficient), n* = 510 (a threshold), and *β* = 30 (the possible maximum lag). Furthermore, the adjusted TOP method and existing TOP are the same for the case of small data (small time-series data size). 

### 2.3. Numerical Tests on Simple Examples 

#### 2.3.1. Comparison on Single-Change-of-Regime in Time Lag

In this subsection, we present the simulation tests of the efficiency of the adjusted TOP approach combining Path Segmentation and Pruning method to detect single and multiple changes of regime and compare the results with the existing TOP method proposed by Sornette and Zhou [7]. First, we consider two stationary time series *X*(*t*_1_),*Y*(*t*_2_) that satisfy the following model:

*Y*(*t*_2_) = 0.8*X*(*t*_2_ − 5) + *η*. (16)

In (16), the time lag between the two time series is 5, i.e., *Y*(*t*_2_) is behind *X*(*t*_1_) at 5 time lag; *η*, denoted as noise, is serially uncorrelated and follows normal distribution with mean of 0 and variance of 0.1. Time series *X*(*t*_1_) itself is generated from an auto-regression process:

*X*(*t*_1_) = 0.7*X*(*t*_1_ − 1) + *ξ*, (17)

where noise *ξ* is serially uncorrelated and follows normal distribution with mean of 0 and variance of 1. In this simulation, we consider time series of duration *N* = 100. For more details about the construction of *X*(*t*_1_),*Y*(*t*_2_), we refer the interested readers to Sornette and Zhou [7].

**Figure 1:**
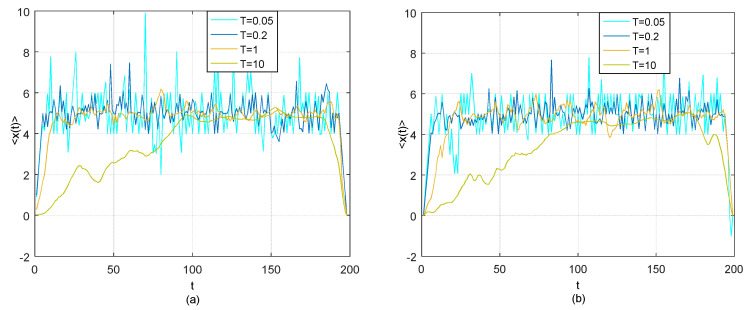
(**a**) Thermal optimal average path (transverse trajectory 〈x(t)〉 as a function of coordinate *t*) for model (16) at *T* = 0.05, 0.2, 1 and 10 based on the existing TOP method; (**b**) Thermal average optimal path 〈x(t)〉 for model (16) at *T* = 0.05, 0.2, 1 and 10 based the adjusted TOP method with *β* = 30.

By using the existing TOP method described as Equations (4)–(9) and the adjusted TOP method described as (15) respectively, the “optimal thermal paths” 〈x(t)〉 can be determined for different temperatures *T* = 1/20, 1/5, 1 and 10. All possible starting positions around origin (0,0) and ending positions around (99,99) are considered for the use of existing TOP method, while the parameter of the maximum time lag *β* is set to be 30 for the use of adjusted TOP method proposed by this paper. Figure 1 shows the transverse trajectory *x* = *t*_2_ − *t*_1_ as a function of the coordinate *t* = *t*_2_ + *t*_1_ based on the existing TOP method and adjusted TOP method. 

It is clear that the results from the two TOP methods are quite similar and the time lag of 5 between the two time series can be discovered by both methods at the relatively low temperature *T* = 1/20, 1/5 and 1.

#### 2.3.2. Comparison on Multiple-Change-of-Regime in Time Lag

We now present the tests of the efficiency of the adjusted TOP method to detect multiple changes of regimes in time lag and compare the results of the existing TOP method. Consider the following model:

(18) $$Y(i)=\{\begin{array}{cc} 0.8X(i)+\eta , & \;1\leq i\leq 50,\\ 0.8X(i-10)+\eta , & \;51\leq i\leq 100,\\ 0.8X(i-5)+\eta , & \;101\leq i\leq 150,\\ 0.8X(i+5)+\eta , & \;151\leq i\leq 200,\\ 0.8X(i)+\eta , & \;201\leq i\leq 250. \end{array}$$

**Figure 2:**
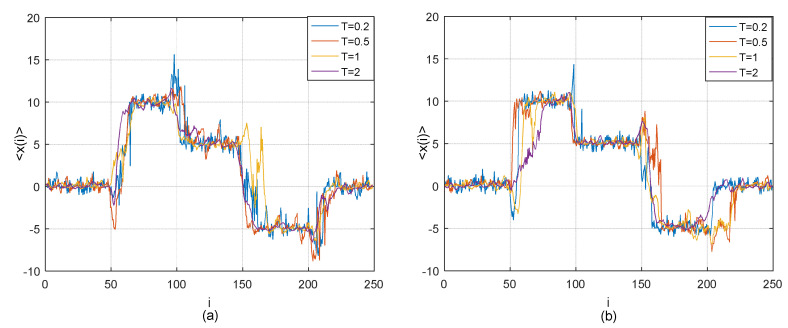
(**a**) Thermal optimal average path (transverse trajectory 〈x(i)〉 as a function of the time step i) for model (18) at four different temperatures *T* = 0.2, 0.5, 1 and 2 based on the existing TOP method; (**b**) Average thermal path (transverse trajectory 〈x(i)〉 as a function of the time step i) for model (18) at four different temperatures *T* = 0.2, 0.5, 1 and 2 based on our adjusted TOP method with *β* = 30.

According to (18), it can be obviously found that the lead-lag relationship between two time series *X*(*t*) and *Y*(*t*) changes in the five different time periods. There is zero time lag between *X* and *Y* in the first and fifth time periods. *Y* is lagging behind *X* with 10 time steps in the second time period. Y is lagging behind *X* with 5 time steps in the third time period, while *Y* is leading before *X* with 5 time steps. The time series *X* is assumed to be the first-order AR process (17) and *η* is a Gaussian white noise with mean of 0 and variance of 0.1. Similarly to Section 2.3.1, by using existing TOP method and adjusted TOP method, we conduct the simulation tests on detection of lead-lag relationship between *X* and *Y* descried as (18) and present the results in Figure 2. 

Figure 2 shows the transverse trajectory 〈x(i)〉 as a function of the time step *i* based on the existing TOP method and adjusted TOP method. It is clear that the results from the two TOP methods are quite similar and the genuine time lag between the two time series in different time period can be successfully identified by both methods at the relatively low temperature *T* = 1/5, although there are short transient crossovers from one time lag to the next at the joint points between the successive time periods.

#### 2.3.3. Comparison on Multiple-Change-of-Regime in Time Lag with Big Data 

(1) *Case 1*. In this case, we expand the whole time interval from [1, 250] to [1, 1250] in order to test the efficiency of the adjusted TOP method in big time-series data. Consider the following model:

(19) $$Y(i)=\{\begin{array}{cc} 0.8X(i)+\eta , & \;1\leq i\leq 250,\\ 0.8X(i-10)+\eta , & \;251\leq i\leq 500,\\ 0.8X(i-5)+\eta , & \;501\leq i\leq 750,\\ 0.8X(i+5)+\eta , & \;751\leq i\leq 1000,\\ 0.8X(i)+\eta , & \;1001\leq i\leq 1250. \end{array}$$

**Figure 3:**
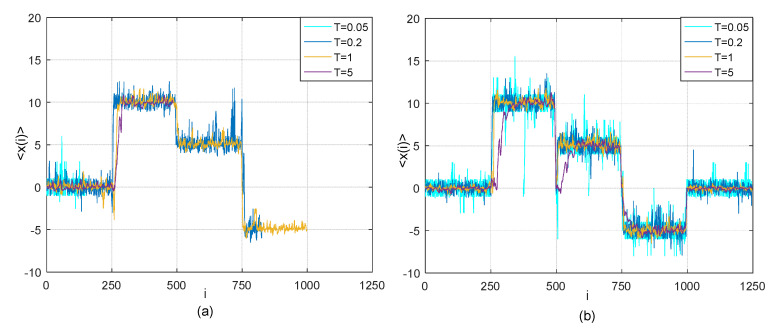
(**a**)Thermal optimal average path (transverse trajectory 〈x(i)〉 as a function of the time step i) for model (19) at four different temperatures *T* = 0.05, 0.2, 1 and 5 based on the existing TOP method; (**b**) Optimal average thermal path (transverse trajectory *x*(*i*) as a function of the time step i) at four different temperatures *T* = 0.05, 0.2, 1 and 5 for model (19) based on our adjusted TOP method with *β* = 30.

According to (19), it can be obviously found that the lead-lag relationship between two time series *X*(*t*) and *Y*(*t*) changes in the five different time periods. There is zero time lag between *X* and *Y* in the first [1, 250] and fifth time periods [1, 1250]. *Y* is lagging behind *X* with 10 time steps in the second time period [251, 500]. *Y* is lagging behind *X* with five time steps in the third time period [501, 750], while *Y* is leading before *X* with 5 time steps [751, 1000]. The time series *X* is assumed to be the first-order AR process (17) and η is a Gaussian white noise with mean of 0 and variance of 0.1. Similarly to Section 2.3.1, by using existing TOP method and adjusted TOP method, we conduct the simulation tests on detection of lead-lag relationship between *X* and *Y* descried as (19) and present the results in Figure 3. 

Figure 3 shows the transverse trajectory 〈x(i)〉 as a function of the time step *i* based on the existing TOP method and adjusted TOP method. For this case, the performances of two methods are obviously different: time lag detection based on the existing TOP method stops at the beginning of the fifth time period as illustrated in Figure 3a, while all genuine time lags between the two time series in different time periods can be successfully recovered by the adjusted TOP method as shown in Figure 3b. In the process of implementation of existing TOP method for this case, we find that the cumulative energy of a thermal path *c*, i.e., cumulative Boltzman factor $G_{⊲}\left(x,t\right)$ gradually increases over time and $G_{⊲}\left(x,t\right)$ becomes unacceptably large before time runs out, thus problem of computational infeasibility takes place.

Moreover, based on the existing TOP method, the performance of test on the detection of time lag between the pairwise has been influenced by temperature *T*. The test may stop earlier at quite low temperature, e.g., *T* = 0.05 or relatively high temperature e.g., *T* = 5, which can be found in Figure 3a. Conversely, by segmenting the whole path into some sub-paths, we use the detailed algorithm illustrated in Algorithm 1 to solve the problem of computational infeasibility and identify all time lags in different time interval finally. It is worth to note that the number of partition of whole thermal path *H*, partition coefficient *n*_1_, threshold n* should be set to be different values under different temperature *T* depending on the testing results from the existing TOP method, when the adjusted TOP method is applied in this case. For example, we make *H* = 2, *n*_1_ = 750, n* = *n*_1_ + *Hβ* = 750 + 2 × 30 = 810 at *T* = 0.2 and 1; and we make *H* = 5, *n*_1_ = 250, n* = *n*_1_ + *Hβ* = 250 + 2 × 30 = 310 at *T* = 0.05 and 5. This is because that the time-series data should be as much as possibly used for accurate detection of changes in time lag before the cumulative Boltzman factor reaching unacceptably large. For more detailed explanation, we refer interested readers to the Appendix. As the results in Figure 3b show, by using the adjusted TOP method, we identify all changes in time lags between the two time series. Therefore, the adjusted TOP method would be more applicable for detection of lead-lag relationship between the two time series with relatively long time duration from this case analysis.

(2) *Case 2*. In this case, we consider two time series X(t) and Y(t) generated from uniform distribution. Their lead-lag relationship is defined as following model:

(20) $$Y(i)=\{\begin{array}{cc} 0.8X(i)+\eta , & \;1\leq i\leq 1000,\\ 0.8X(i-10)+\eta , & \;1001\leq i\leq 2000,\\ 0.8X(i-5)+\eta , & \;2001\leq i\leq 3000,\\ 0.8X(i+5)+\eta , & \;3001\leq i\leq 4000,\\ 0.8X(i)+\eta , & \;4001\leq i\leq 5000. \end{array}$$

**Figure 4:**
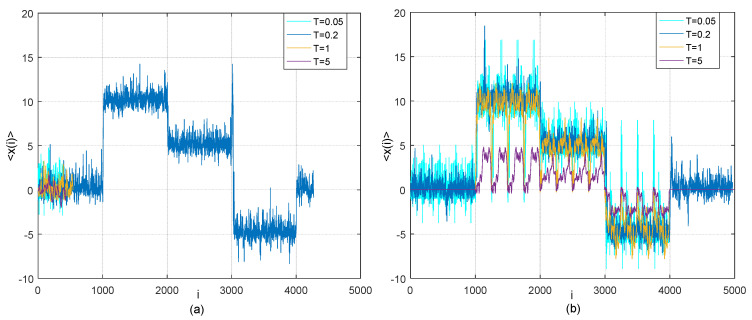
(**a**) Thermal optimal average path (transverse trajectory *x*(*i*) as a function of time step *i*) for model (20) at four different temperatures *T* = 0.05, 0.2, 1 and 5 based on the existing TOP method; (**b**) Average thermal path (transverse trajectory *x*(*i*) as a function of time step *i*) for model (20) at four different temperatures *T* = 0.05, 0.2, 1 and 5 based on our adjusted TOP method with *β* = 30.

In (20), it can be obviously found that the lead-lag relationship between two time series *X*(*t*) and *Y*(*t*) changes in the five different time periods. There is zero time lag between *X* and *Y* in the first [1, 1000] and fifth time periods [4001, 5000]. *Y* is lagging behind X with 10 time steps in the second time period [1001, 2000]. *Y* is lagging behind *X* with 5 time steps in the third time period [2001, 3000], while *Y* is leading before *X* with 5 time steps [3001, 4000]. The differences between Case 1 and Case 2 are that (1) the time period is expanded from [1, 1250] in Case 1 to [1, 5000] in Case 2; (2) the time series *X*(*t*) is assumed to follow a uniform distribution in the interval (0, 1) for Case 2. Similarly, by using existing TOP method and adjusted TOP method, we conduct the simulation tests on detection of lead-lag relationship between *X* and *Y* descried as (19) and present the results in Figure 4.

Figure 4 shows the different performance of the two methods for testing the time lag between two time series in different time periods. All time lags are not successfully identified by existing TOP method; and time lag between *X* and *Y* in the first time period cannot even be detected by existing TOP method with temperature *T* = 0.05, 1 and 5, as illustrated in Figure 4a. Conversely, all genuine time lags between the two time series in different time periods can be successfully recovered by the adjusted TOP method as shown in Figure 4b. 

In summary, from the above comparative analyses we conclude that both the existing TOP method and the adjusted TOP method can be applied to detect the lead-lag relationship between two time series when the time-series data size is relatively small (see Section 2.3.1 and Section 2.3.2), while the adjusted TOP method outperforms the existing TOP method for recovering time lag between two time series when the time-series data size is relatively big (see Section 2.3.3). It worthwhile to note that the existing TOP method should be considered before implementing the adjusted TOP method for the purpose of achieving reasonable setting of the adjusted TOP method’s parameters such as the number of partition of whole thermal path *H*, partition coefficient *n*_1_ and threshold n* (see Section 2.3.3 Case 1). The reasoning is that although the existing TOP method is not applicable in case of big time series data, it can provide the reference values of the parameters for investigators. The settings of these parameters associate with the length of the considered time series as well as the computer storage and operation speed. The algorithm or theoretical analysis for the optimal settings of these parameters certainly requires in the future studies.

## 3. Empirical Results

### 3.1. Data Source and Description

**Figure 5:**
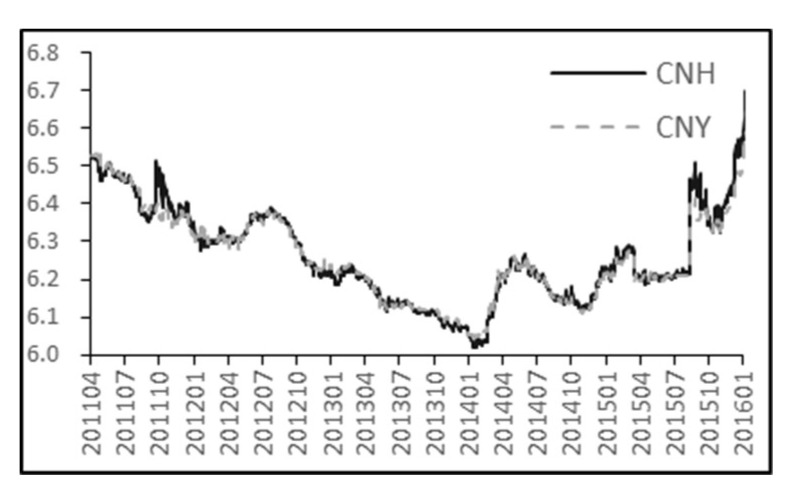
Closing price of CNY and CNH spot.

Daily exchange rate data for the onshore spot (CNY) and offshore spot (CNH) markets are from Bloomberg. We use USD/CNY and USD/CNH exchange rate series from April 18, 2011 to January 29, 2016. For studying the relationship between onshore spot and offshore spot, we use CNY /CNH pairwise data. We discard the weekends and holidays during the observation period, because there is virtually no foreign exchange trading during these days. Finally, total 1250 groups of data are obtained. Each group of data includes: the daily highest price, lowest price and closing price of USD / CNY and USD / CNH exchange rate. For convenience, in the following content USD / CNH and USD / CNY spot exchange rate are noted as CNH and CNY respectively. The changing trends of closing price of CNH and CNY spot are present in Figure 5 below. The notations used in Section 3 and their explanations are shown in Table 1. 

### 3.2. Liquidity Measure

Exchange rate bid-ask spread (BAS) has been widely used to reflect the liquidity level of a FX market due to its effectiveness [1]. In this paper, we employ a simple method proposed by Corwin and Schultz [26] to estimate BAS of CNH and CNY. This method uses daily highest and lowest price for estimating BAS. More specifically, the method uses high-low price ratio to reflect BAS and provides a high-low spread estimator. The main ideas behind this method can be described by the following equations:

(21) $$S_{t}=\frac{2\left(e^{\alpha_{t}}-1\right)}{1+e^{\alpha_{t}}},\;t=1,2,\ldots ,\;n-1,$$

(22) $$\alpha_{t}=\frac{\sqrt{2\beta }-\sqrt{\beta }}{3-2\sqrt{2}}-\sqrt{\frac{\gamma_{t}}{3-2\sqrt{2}}},\;t=1,2,\ldots ,\;n-1,$$

(23) $$\beta =\frac{1}{n-1}\sum_{t=1}^{n-1}\left\{\sum_{j=0}^{1}{\left[ln\left(\frac{H_{t+j}}{L_{t+j}}\right)\right]}^{2}\right\},$$

(24) $$\gamma_{t}={\left[ln\left(\frac{H_{t,t+1}}{L_{t,t+1}}\right)\right]}^{2},t=1,2,\ldots ,\;n-1.$$

**Figure 6:**
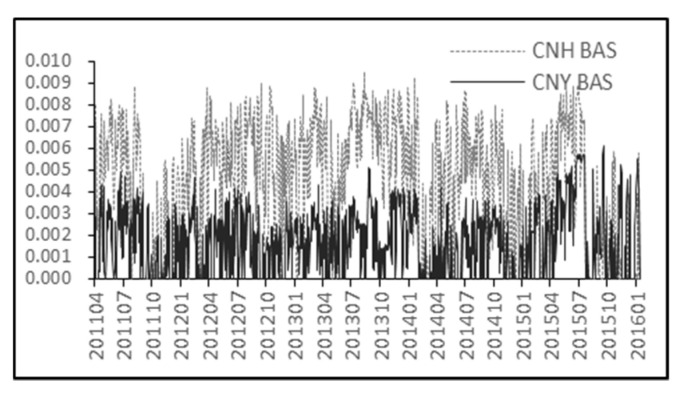
CNY and CNH Bid-Ask spread.

The detailed description of variables in Equations (21)–(24) can be found in Algorithm 1. Since both onshore spot (CNY) and offshore spot (CNH) high-low spread are computed by (21)–(24), we drop the superscripts of the variables in (21)–(24) for convenience. $\gamma_{t}$ are computed by squaring natural log of the ratio of high to low price over two consecutive single days. According to (21)–(24), we compute the CNY (CNH) BAS and their statistics such as mean and standard deviation etc. that can be found in Table 2. We use Figure 6 to show the change trend of CNH and CNY spot BAS during the observation period. 

The results in both Table 2 and Figure 6 show that CNH BAS is higher than CNY BAS which implies relatively poor liquidity of CNH market compared with CNY market. However, the difference between CNH BAS and CNY BAS exhibits an obvious downward trend in most recent years especially after the first half of 2015. This phenomenon is likely due to a series of steps such as creating a whole slate of investment products for qualified foreign institutional investors, reducing the Yuan clearing interest rate and easing the restriction on trade settlement that can be seen as improving CNH spot liquidity (see Ding [1] and Cushnie [21]).

### 3.3. Preliminary Analysis by Vector Auto-Regression Model

The vector auto-regression (VAR) model is typical conventional method used to detect the causality relation between two time series. VAR method has been used to investigate the relationship between onshore CNY spot and offshore non-deliverable forward rate [1]. In this subsection, as a comparison study of the CNY spot and CNH spot with following results from thermal optimal path method, we use VAR model to perform a causality analysis by using all data from April 18, 2011 till January 29, 2016. 

The daily closing price series $Cls_{t}^{CNY}$ and $Cls_{t}^{CNH}$, the first difference series $d_{t}^{CNY}=Cls_{t+1}^{CNY}-Cls_{t}^{CNY}$ and $d_{t}^{CNH}=Cls_{t+1}^{CNH}-Cls_{t}^{CNH}$ (*t* = 1,2,…,*n* − 1), and BAS series $S_{t}^{CNY}$ and $S_{t}^{CNH}$ are taken to test for the stationarity of the data sets before the causality test is implemented. Augmented Dickey-Fuller (ADF) test method is used and the results of these tests are summarized in Table 3. The tests for $Cls_{t}^{CNY}$ and $Cls_{t}^{CNH}$ provide no evidence against the unit root null hypothesis, thus both daily closing price series of offshore CNH spot and onshore CNY spot, $Cls_{t}^{CNH}$ and $Cls_{t}^{CNY}$ are non-stationary. Conversely, the results show that the other four time series are significantly stationary at 1 percent level. 

The following vector auto-regression (VAR) models are used to implement causality test provided that the first difference series and BAS series are stationary:

(25) $$d_{t}^{CNY}=\alpha_{0,1}+\sum_{i=1}^{p}\alpha_{i,1}d_{t-i}^{CNY}+\sum_{j=1}^{q}\beta_{j,1}d_{t-j}^{CNH}+\varepsilon_{1,t},$$

(26) $$d_{t}^{CNH}=\alpha_{0,2}+\sum_{i=1}^{p}\alpha_{i,2}d_{t-i}^{CNH}+\sum_{j=1}^{q}\beta_{j,2}d_{t-j}^{CNY}+\varepsilon_{2,t},$$

(27) $$S_{t}^{CNY}=\alpha_{0,3}+\sum_{i=1}^{p}\alpha_{i,3}S_{t-i}^{CNY}+\sum_{j=1}^{q}\beta_{j,3}S_{t-j}^{CNH}+\varepsilon_{3,t},$$

(28) $$S_{t}^{CNH}=\alpha_{0,4}+\sum_{i=1}^{p}\alpha_{i,4}S_{t-i}^{CNH}+\sum_{j=1}^{q}\beta_{j,4}S_{t-j}^{CNY}+\varepsilon_{4,t},$$

where *ε_k,t_*(*k* = 1,2,3,4) is the white noise. Following the spirit of Ding et al. [1], for each VAR function above, we use five lags, i.e., *p = q* = 5, given one week of trading activity. Table 4 and Table 5 show the test results of 4 VAR models. The results in Table 4 indicate that the overall regression models are significant at 10% significance level. This implies that the changes in CNY spot cause the changes in CNH spot while the changes in CNH spot cause the changes in CNY spot during the observation period. The causality exists between CNY Bid-ask spread and CNH Bid-ask spread also. 

Table 5 presents the coefficient estimate results of models (25)–(28). The values in the 2nd and 3rd columns are the estimation and *p*-value of coefficients of regression function (25). The values in other columns are the coefficients estimation of models (26)–(28). These results in Table 5 also reflect that onshore CNY spot market and offshore CNH spot market have a bidirectional relationship from two aspects of daily closing price change and liquidity. Taking the results of regression functions (25) and (26) as an example, besides the own influence, the difference of closing price of CNY spot rate at *t*-th day, $d_{t}^{CNY}$. is significantly influenced by the difference of closing price of CNH spot rate at (*t* − 1)-th day and (*t* − 2)-th day, i.e., $d_{t-1}^{CNH}$ and $d_{t-1}^{CNH}$. In turn, the difference of closing price of CNH spot rate at *t*-th day, $d_{t}^{CNH}$ is significantly influenced by the difference of closing price of CNY spot rate at (*t* − 1)-th day, $d_{t-1}^{CNY}$.

The causality test based on VAR method above indicates that CNH and CNY have lagged impact on each other in the short term price change process. The CNY spot rate today is influenced by CNH spot at previous one day or two days, while CNH spot rate today is influenced by CNY spot rate on the previous day (In the preliminary analyses of relationship between CNH and CNY spot, for simplicity, we only consider the linear causality test based on vector auto-regression model. The parametric nonlinear relations test for foreign exchange rates time series has been considered in the literature (see e.g., Ma and Kanas [27]; Bekiros and Diks [28]), but it is out of the scope of this study). However, the result obtained by this method cannot tell us who is “the leader” and who is “the follower” obviously. Also, these results cannot address the question how the lead-lag relation between CNY/CNH pair changes over the different time periods, as economic conditions change. Below, with the non-parametric method of thermal optimal causality path, we try to answer these problems bypassed by existing causality analysis based on VAR model. 

### 3.4. Analysis on Results of Thermal Optimal Causality Paths

#### 3.4.1. The Results from Adjusted TOP Method

**Figure 7:**
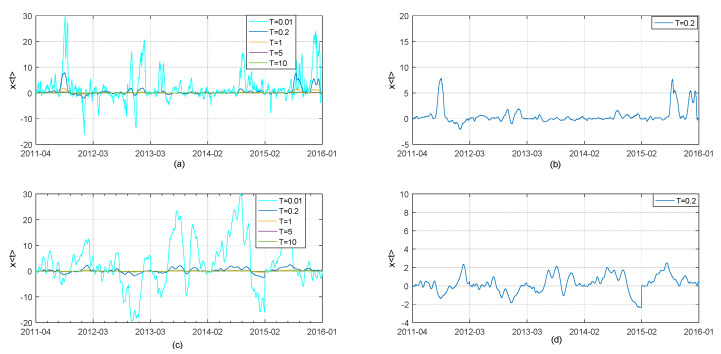
Thermal optimal average paths of CNH and CNY spot rate and BAS with partition coefficient *n*_1_ = 420, the threshold n* = 510, the possible maximum lag *β* = 30 at different temperatures. Chart (**a**): Thermal optimal average paths (transverse trajectory *x* = *t*_2_ − *t*_1_ as a function of observation time) between the first difference series of CNH daily closing price (described as *X*(*t*_1_)) and the first difference series of CNY daily closing price (described as Y(*t*_2_)) at temperature T = 0.01, 0.2, 1, 5 and 10; Chart (**b**): Thermal optimal average path between the first difference series of CNH daily closing price and the first difference series of CNY daily closing price at temperature T = 0.2; Chart (**c**): Thermal optimal average paths (transverse trajectory *x* = *t*_2_ − *t*_1_ as a function of observation time) between CNH BAS (described as *X*(*t*_1_)) and CNY BAS series (described as Y(*t*_2_)) at temperature T = 0.01, 0.2, 1, 5 and 10; Chart (**d**): Thermal optimal average path between CNH BAS and CNY BAS series at temperature T = 0.2.

We here also use the stationary first difference series of closing price of CNH /CNY spot $d_{t}^{CNY}$, $d_{t}^{CNH}$ and CNH/CNY BAS series $S_{t}^{CNY}$, $S_{t}^{CNH}$ spanning from April 18, 2011 to January 19, 2016 to implement the adjusted TOP method described in Section 2. We obtain the thermal optimal causality paths 〈x(t)〉 between the first difference series of closing price of CNH and CNY spot at different temperatures. The graph of 〈x(t)〉 is shown in upper left of Figure 7. 

We assume that *X*(*t*_1_) and *Y*(*t*_2_) represent CNH time series and CNY series respectively (recall Section 2.1). Therefore, according to the previous analysis in Section 2.1, positive *x* = *t*_2_ − *t*_1_ means that CNH spot guides CNY spot, i.e., changes in closing price of CNH spot rate happened before similar changes happened in CNY spot market. Conversely, negative *x* means that CNY spot guides CNH spot, i.e., changes in closing price of CNY spot rate happened before similar changes happened in CNH spot market. Finally, if *x* equals to 0 or changes slightly around 0, it means that CNH and CNY react upon one another without obvious lead-lag relationship. 

The horizontal and vertical axes of “chart (a)” in the upper left part of Figure 7 correspond to the observation period and the thermal optimal path between difference series of closing price of the CNH and CNY spot markets, respectively. “*T*” corresponds to the temperature. “Chart (a)” shows that the thermal optimal path becomes increasingly sharp with temperature *T* decreasing, because the influence of noise is quite large for the small *T*, e.g., the case of *T*= 0.01. On the contrary, the curve of 〈x(t)〉 is so flat that noise and some probably important information are averaged out when the temperature is quite high, e.g., the case of *T* = 10. These results are consistent with the previous analysis on parameter T of TOP method. We select the thermal optimal path of *T* = 0.2 for further analysis, since the curve 〈x(t)〉 at *T* = 0.2 is neither too sharp nor too flat. We present it individually in “chart (b)” in the upper right of Figure 7. Besides, the thermal optimal causality paths 〈x(t)〉 between Bid-Ask spread of CNH spot and Bid-Ask spread of CNY spot series at different temperature is shown in “chart (c)” at bottom left of Figure 7. The fluctuation of the graph of TOP 〈x(t)〉 in chart C is increasingly vigorous as the temperature *T* decreases, which is similar to results presented by “chart (a)”. For further analysis, we also select the case of thermal optimal path of *T* = 0.2 and present it in “chart (d)” at the bottom right of Figure 7.

Chart (b) in Figure 7 indicates that lead-lag relationship between CNH/CNY pair changes for different time periods. Chart (b) shows that the graph of thermal optimal path 〈x(t)〉 changes slightly around 0 most of time during almost 5-year period of observation. This means that CNH spot and CNY spot react basically upon one another without obvious lead-lag relationship. The obvious deviations from 0 occur on the third quarter of 2011 and second half of 2015. It is well-known that both U.S.A and Europe experienced a significant drop in stock prices in August of 2011. The big drop in stock prices was perceived as a sign of economy going down. On the other hand, the People’s Bank of China announced reform of the RMB mid-price quotation process in August 2015, improving RMB exchange rate formation mechanism but causing simultaneously enormous fluctuation of the RMB value at that time. Subsequently, the TED spread was exploding at the end of December, 2015, signaling an increasing level of risk in the financial markets. We divide the observation period into two phases in this paper. The period of time when a significant economic event influencing FX market occurs is defined as a special phase. Thus third quarter of 2011 and second half of 2015 can be said to be a special phase. The period of time when the world financial market is relatively stable is defined as the normal phase.

Results in chart (b) indicate that a significant difference between CNH closing and CNY closing exists during the special phase; furthermore, changes in offshore CNH spot happened before onshore CNY spot changing since 〈x(t)〉 is significantly greater than 0. In other words, offshore CNH spot market may react more quickly to the changes of international market or the reform of RMB exchange rate formation mechanism compared with onshore CNY spot market. The change of onshore CNY spot tracks and follows the changes of offshore CNH spot price for a while until this lead-lag relationship disappears. From this perspective, offshore CNH spot market dominates onshore CNY spot market in turbulent and highly uncertain economic conditions. The changing tendency of price presented in Figure 5 also gives a hint that the deviation between closing price of CNH and CNY spot is quite obvious in the third quarter of 2011 and second half of 2015, while closing price of CNH spot seems to synchronize with CNY spot during the rest of observation period. However, from chart (b), we do not find the evidence that a lasting and stable guidance of offshore CNH spot market on onshore CNY spot market exists at the special stage, since the thermal optimal path function 〈x(t)〉 does not provide a stable value of lag. This may be because tradeoff between the influence of offshore CNH spot market on onshore CNY spot market and the influence of the regulation from the People’s Bank of China on onshore CNY spot market and there is need for further study. 

On another front, results on CNH and CNY market liquidity in “Chart (d)” present that the thermal path 〈x(t)〉 is almost between -2(day) and 2(day) around the time axis without violent fluctuations seen in the special phase, unlike the results in “Chart (b)”. This means that changes in CNH market liquidity occur sometime, while the changes in CNH market liquidity lag behind the change in CNY market liquidity. The results based on the TOP method furthermore indicate that there is no obvious lead-lag relation between CNH and CNY market liquidity during the observation period. In general, CNH market liquidity reacts to the changes in economic conditions earlier, compared with CNY market due to positive value of 〈x(t)〉 most of time during the observation period. However, the stable lead-lag relation is not formulated, which may be caused by the strict restrictions on CNH market liquidity from PBC and the HKMA.

#### 3.4.2. Analysis on Influence of Maximum Lag Results on Thermal Optimal Path

**Figure 8:**
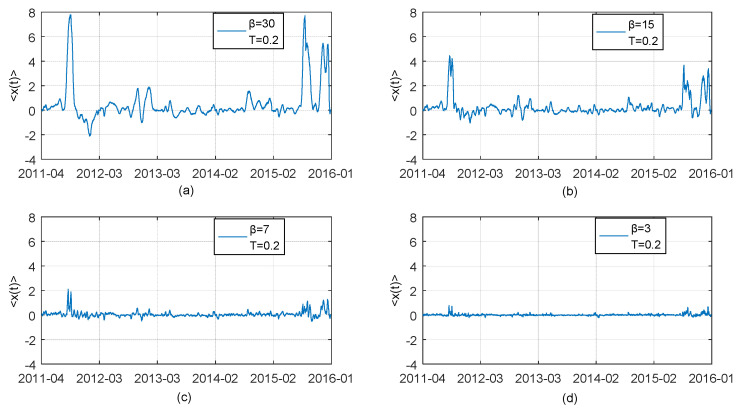
Thermal optimal average paths (transverse trajectory *x* = *t*_2_ − *t*_1_ as a function of observation time) between the first difference series of CNH daily closing price (described as *X*(*t*_1_)) and the first difference series of CNY daily closing price (described as *Y*(*t*_2_)) obtained by using adjusted TOP method with four different assumed maximum lag *β* = 30,15,7,3. Chart (**a**): The case for *β* = 30; Chart (**b**): The case for *β* = 15; Chart (**c**): The case for *β* = 7; Chart (**d**): The case for *β* = 3.

**Figure 9:**
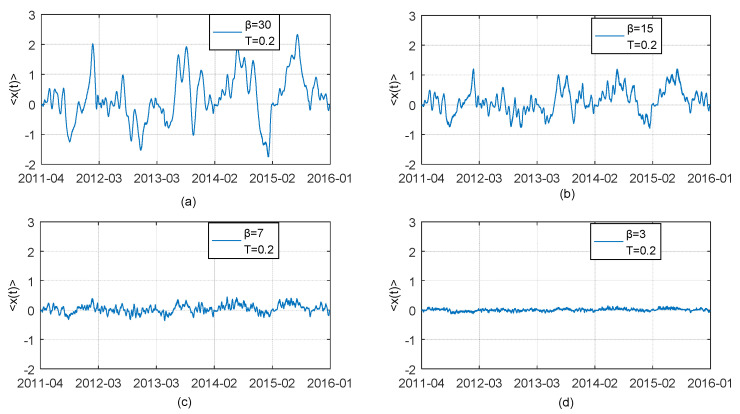
Thermal optimal average paths (transverse trajectory *x* = *t*_2_ − *t*_1_ as a function of observation time) between CNH BAS (described as *X*(*t*_1_)) and CNY BAS series (described as *Y*(*t*_2_)) obtained by using adjusted TOP method with four different assumed maximum lag *β* = 30,15,7,3. Chart (**a**): The case for *β* = 30; Chart (**b**): The case for *β* = 15; Chart (**c**): The case for *β* = 7; Chart (**d**): The case for *β* = 3.

In our study, we propose the Pruning and Path Segmentation algorithm (PPSA) to fully address the problem of computational infeasibility of the original TOP method shown in Section 2.2, when the cumulative Boltzman factors involved tend to become unacceptably large with increasing sample size. Naturally, *β*, maximum lag is an important parameter for PPSA and should be predetermined by ones. If different values of maximum lag *β* bring about dramatically changes of results even opposite results and conclusion, this means that PPSA may not perform steadily. Therefore, in this part, we show the influence of changes in maximum lag *β* on the results of the average optimal thermal paths. We set *β* = 30, 15, 7, 3 respectively and obtain an average optimal thermal paths between difference series of CNH spot rate and CNY spot rate. We present the corresponding results by Charts (a), (b), (c), (d) in Figure 8. Similarly, we present the results of average optimal thermal paths between CNH and CNY Bid-Ask Spread series by Charts (a), (b), (c), (d) in Figure 9. The results show that whole tendency of TOP path does not significantly change as *β* changes, when the value of *β* is set to be relatively large. Although the value of 〈x(t)〉 is reducing with *β* decreasing, features of lead-lag over the period of observation depicted by average thermal optimal paths remain almost the same. This indicates that the results and related analyses based on PPSA are reliable. Meanwhile TOP method’s popularity would be enhanced due to introduction of PPSA to some extent. It is worthwhile to note that quite small values of *β*, e.g., *β* = 3 (see the case of Figure 8d), are not suitable, because too small *β* provides little information for the detection of lagged dependency relation between two time series.

## 4. Conclusions

The foreign exchange price change and liquidity should be the two important indicators that draw concern from investors and policy makers. In this paper, we study the correlation between the CNH spot market and CNY spot market from the two aspects of daily closing price change and Bid-Ask spread (BAS) as a proxy of market liquidity. 

By using vector auto-regression model, we find that the causality exists between both intraday price changes for CNY/CNH spot rate and CNH/CNY BAS during the observation period of 2011–2016. Furthermore, by using the adjusted thermal optimal method, we also detect the local lead-lag dependence between CNH and CNY spot market in the contexts of daily closing price change and Bid-Ask spread (2011–2016). The results show that offshore CNH market plays the role of a Forerunner, i.e., the changes in offshore CNH spot rates precede the changes in onshore CNY spot rates, while onshore CNY spot plays the role of a Follower, at the beginning of economic turmoil. The increasingly strong influence of onshore CNY on offshore CNH leads to the disappearance of the lead-lag relationship between CNH/CNY pair gradually. On the other hand, the local lead-lag correlation between CNH/CNY BAS (liquidity) cannot be discovered, unlike daily closing price change of CNH and CNY spot. Neither the Forerunner nor the Follower is obvious, even when economic conditions change. The strict restriction on cross-border cash flow would be the reason why the lead-lag relationship between CNH market liquidity and CNY market liquidity is not found. Difference in determinants that influence the foreign exchange price formulation and liquidity would be the essential reason. 

Our studies may help both policy makers and investors to make decision from the perspective of application. An investor can understand the market liquidity situation and get better returns via observing the pricing signals from the offshore/onshore market over the different time periods. For example, the investor may efficiently predict the changes in CNY spot rate according to the changes occurring in CNH spot rate market and hedge the risk associated with trading foreign currency, especially when the economic conditions begin to change obviously. For policymakers, Chinese government may need to consider the linkage between CNH and CNY spot markets and the possible policy transmission when constructing regulatory policy of renminbi foreign exchange market. Further, the government may take measures for relaxing the restriction of liquidity of offshore exchange rate market, such as permitting cross-border renminbi funds flowing from the onshore to the offshore market. In this way, the linkage between onshore and offshore markets can be strengthened, and the adverse effect of international economic pressure on offshore market can be compensated, thereby stabilizing the renminbi exchange rate and promoting the development of the market.

Last but not least, we propose an algorithm combining Pruning and Path segmentation to overcome the difficulty of large-scale computation required in thermal optimal path function involving cumulative Boltzman factors and improve the performance of existing TOP method in the application of the detection of long-range lagged dependence between two time series. Moreover, the theoretical analyses and simulation results are presented to verify validity of the adjusted thermal optimal path method in the setting of big time-series data set. Still, despite all this, the efficiency, effectiveness and robustness of adjusted thermal optimal path method for use in other application areas may need to further study. Besides, the dependence between CNH/CNY spot rates series may need further investigation by other more methods such as graph theory approach, complex network and deep learning approach. How to appropriately use them is still an interesting problem which requires detailed work.

## Figures and Tables

**Table 1:** Model notation.

Notation	Explanation
$Cls_{t}^{CNY}$	USD / CNY closing price on day *t*, *t* = 0,1,2,…,*n*
$Cls_{t}^{CNH}$	USD / CNH closing price on day *t*, *t* = 0,1,2,…,*n*
$H_{t,t+1}^{CNY}$	the highest USD / CNY spot rate over the two days *t* and *t* + 1, *t* = 0,1,2,…,*n* − 1
$L_{t,t+1}^{CNH}$	the lowest USD / CNH spot rate over the two days *t* and *t* + 1, *t* = 0,1,2,…,*n* − 1
$S_{t}^{CNY}$	the spread between bid and ask price of CNY spot on day *t*, *t* = 0,1,2,…,*n* − 1
$S_{t}^{CNH}$	the spread between bid and ask price of CNH spot on day *t*, *t* = 0,1,2,…,*n* − 1

**Note**: in Table 1 time *t* = 0 and *t* = 1 corresponds to April 18, 2011 and April 19, 2011 respectively, and so on, *t* = 1249 corresponds to January 29, 2016.

**Table 2:** Descriptive Statistics of CNY bid-ask spread (BAS) and CNH BAS.

Variable	Mean	Stand Deviation	Maximum	Minimum
$S_{t}^{CNY}$	0.0017	0.0015	0.0061	0
$S_{t}^{CNH}$	0.0044	0.0028	0.0095	0

**Table 3:** Augmented Dickey-Fuller test for stationarity.

H_0_: Time Series Has a Unit Root; H_1_: Time Series Is Stationary		
Time Series	ADF Statistic	*p*-Value
$Cls_{t}^{CNY}$	0.17939	0.99
$Cls_{t}^{CNH}$	−0.30993	0.99
$d_{t}^{CNY}$	−9.7336	0.01 ***
$d_{t}^{CNH}$	−10.797	0.01 ***
$S_{t}^{CNY}$	−6.8676	0.01 ***
$S_{t}^{CNH}$	−4.5958	0.01 ***

Note: in Table 3 lower amount of *p*-value for a time series presents that the time series is stationary from statistical perspective. Furthermore, “***” indicates that the alternative hypothesis of the corresponding time series being stationary is accepted at significance level of 0.01.

**Table 4:** Causality test.

Null Hypothesis H_0_	$d_{t}^{CNY}$ Does Not Cause $d_{t}^{CNH}$	$d_{t}^{CNH}$ Does Not Cause $d_{t}^{CNY}$	CNY BAS Does Not Cause CNHBAS	CNH BAS Does Not Cause CNYBAS
F-Statistic	2.13577	8.48935	1.93456	3.20295
*p*-Value	0.0589	7E−08	0.0859	0.0070
Test result	Reject H_0_	Reject H_0_	Reject H_0_	Reject H_0_

**Table 5:** Estimations for the VAR models (25)–(28).

Parameter	$d_{t}^{CNY}$		$d_{t}^{CNH}$		$S_{t}^{CNY}$		$S_{t}^{CNH}$	
	Estimation *p*-Value		Estimation *p*-Value		Estimation *p*-Value		Estimation *p*-Value	
*α*_0_	3.3E−05	0.4408	0.0001	0.3814	0.0003	3.4E−06	0.0007	3.48E−11
*α*_1_	−0.1522	0.0792 *	−0.0505	0.0875	0.5133	6E−58 ***	0.7191	6.67E−102
*α*_2_	−0.1319	0.0002 ***	−0.0177	0.0203	0.0430	0.1356	−0.0845	0.0114
*α*_3_	0.0128	0.3660	−0.1474	4.83E−5 ***	0.0034	0.4602	0.1158	0.0009 ***
*α*_4_	−0.0281	0.2227	−0.0984	0.0047	0.0734	0.0158	0.0123	0.3697
*α5*	0.0217	0.2738	0.0009	0.4903	0.0948	0.0010	0.0782	0.0053
*β1*	0.1380	1.5E−8 ***	0.1522	0.0032 ***	0.0732	9E−05 ***	0.0404	0.1986
*β2*	0.1014	3.1E−5 ***	0.0690	0.2468	−0.0349	0.0698	−0.0557	0.0060 ***
*β3*	0.0047	0.4253	0.0385	0.0517	0.0048	0.4197	−0.1001	0.0309
*β4*	0.0260	0.1506	0.0905	0.0517	−0.0183	0.2190	0.1346	0.1488
*β5*	0.0475	0.0283	0.0134	0.4026	0.0073	0.3534	−0.0427	0.1871

Note: in Table 5 lower amount of *p*-value for a factor presents that the factor has significant effect on response. “*” and “***” indicate that the corresponding factors influence the response at significance levels of 0.1 and 0.01 respectively.

## Appendix A

This part, we illustrate how to use Path Segmentation method to determine the average thermal optimal path 〈x(t)〉 when related *g*(*i*, *t* − *i*) are unacceptably large. Let us define some notations firstly.

Let *n** be a threshold. Suppose for both *i* and *j* not greater than *n**, the cumulative Boltzman factor *g*(*i*,*j*) can be obtained; otherwise the value of *g*(*i*,*j*) becomes unacceptable large. It is not difficult to determine the suitable value of *n** with the related empirical results for any specific cases.

Let *n* be the maximum subscription of data. Since we denote the first data pair as (*x*_0_, *y*_0_), the sample size is *n* + 1. We divide all samples into *H* groups. The number of the groups and the number of sample of each group would not unique. In our case, we divide all samples into almost equally 3 groups with consideration of convenience of calculation i.e., *H* = 3. The first group consists of *n*_1_ + 1 data. The second group consists of *n*_1_ data; and the third one consists of *n* − 2*n*_1_. We define *n*_1_ as partition coefficient in this paper. Once *H* is set to 3, then we request that 2*n*_1_ < *n* ≤ 3*n*_1_; *n** ≥ *n*_1_ + 3*β*; and *n*_1_ > 2*β*, where *β,* a positive integer, representing the possible maximum lag. For the general case, the request becomes: (*H* − 1)*n*_1_ < *n* ≤ *Hn*_1_, *n** ≥ *n*_1_ + *H**β*, and *n*_1_ > (*H* − 1)*β*. These requirements about *n*_1_, *n** and *β* can be met easily, because the way to divide all samples is not unique. Furthermore, in order to use as much data as possible and therefore use more information from data, we can set *n*_1_ to be minimum of integer value of *n*/2 and *n** − 3*β*. In this study, *n* = 1249 and we set *n*_1_ = 420, *n** = 510, and *β* = 30.

Now, we present the process of determination of 〈x(t)〉 that involves the computation of *g*(*i*,*j*) for *i*, *j* subject to *n** < *i*, *j* < *n** + *n*_1_ − *β*. It is obviously infeasible to directly compute the value of *g*(*i*,*j*) when both *i*,*j* > *n**. In our case, we find that *g*(*i*,*j*) is still not computable when *I* > *n** and 0 < *j* ≤ *n** or *j* > *n** and 0 < *i* ≤ *n**. We will talk about this later. Let us go back to the case when *n** < *i*,*j* < *n** + *n*_1_ −*β*. Furthermore, we have that *i*,*j* > *n*_1_ + *β* is satisfied, because of the predetermined assumption of *n** ≥ *n*_1_ + 3*β*.

It is easily to be known that any path from origin point (0,0) to ending point (*i*,*j*) must pass through a point among 2*β* + 1 points satisfying {(*t*_1_,*t*_2_): *t*_1_ = *n*_1_, *n*_1_ − *β*≤ *t*_2_ ≤ *n*_1_ + *β*, *t*_1_ and *t*_2_ are integers}, because all paths must lie between −*β* ≤ *t*_2_ − *t*_1_ ≤ *β* under the assumption of (14) and *i*, *j* > *n** ≥ *n*_1_ + 3*β*. Thus, all paths from origin to (*i*,*j*) can be described by a union of 2*β* + 1 disjoint path sets passing through the different 2*β* + 1 points, i.e., (*n*_1,_ *n*_1_ − *β*), (*n*_1_,*n*_1_ – *β* + 1),…,(*n*_1_, *n*_1_ + *β*).

Let *P_K_*_+1_ represent a path set defined as follows:

*P_K_*_+1_ = $P_{K+1}^{\left(1\right)}$ + $P_{K+1}^{\left(2\right)}$ = {*C*^(1)^: *C*^(1)^ = *C*_(0,0)__→(*n*1, *n*1-_*_β_*_+*K*)_} +{*C*^(2)^: *C*^(2)^ = *C*_(*n*1, *n*1−_*_β_*_+*K*)__→(*i*,*j*)_}

= {*C*: *C* = *C*^(1)^ + *C*^(2)^}, *K* = 0,1,…,2*β*.

Here *C*_(0,0)__→(*n*1, *n*1−_*_β_*_+K)_ represents a path starting from (0,0) and ending at (*n*_1_,*n*_1_ − *β* + *K*). Moreover, there is only point of intersection of the path denoted by *C*_(0,0)__→(*n*1, *n*1 −_ *_β_*_+ K)_ and vertical line *t*_1_ = *n*_1_ in coordinates (*t*_1_, *t*_2_). Here, *C*_(*n*1, *n*1 − β__+ *K*)__→(*i*,*j*)_ represents an arbitrary path starting from (*n*_1_,*n*_1 −_ *β* + *K*) ending at (*i*,*j*). *C* is a path from origin (0,0) to (*i*,*j*). Thus, the union of *P_K_*_+1_, *K* = 0,1,…,2*β*, is the set of all path starting from (0,0) and ending at (*i*,*j*) and these path sets denoted by *P_K_*_+1_ are disjoint. “Disjoint path set” here means that there is no any completely same path in different path sets *P_K_*_+1_ with different value of *K*. We discuss how to obtain the cumulative Boltzman factors of different path sets *P_K_*_+1_ below.

① For *C*∈P_1_(*K* = 0), *C* can be divided into two segments denoted by *C*^(1)^ and *C*^(2)^, i.e., *C* =*C*^(1)^ + *C*^(2)^ = *C*_(0,0)__→(*n*1, *n*1 −_ *_β_*_)_ + *C*_(*n*1, *n*1 −_ *_β_*_)__→(*i*,*j*)._ The energy corresponding *C* can be described by:

(A1) $$E_{C}=E_{C^{\left(1\right)}}+E_{C^{\left(2\right)}}-\varepsilon (n_{1},n_{1}-\beta ).$$

So, the Boltzmann factor corresponding to path C is:

(A2) $$e^{-E_{c}}=e^{-E_{C^{\left(1\right)}}}\cdot e^{-E_{C^{\left(2\right)}}}\cdot e^{\varepsilon (n_{1},n_{1}-\beta )}.\;$$

Furthermore, we have:

(A3) $$\sum_{C\in P_{1}}e^{-E_{C}}=\sum_{C^{\left(1\right)}\in P_{1}^{\left(1\right)}}\;e^{-E_{C^{\left(1\right)}}}\cdot \sum_{C^{\left(2\right)}\in P_{1}^{\left(2\right)}}\;e^{-E_{C^{\left(2\right)}}}\cdot e^{\varepsilon (n_{1},n_{1}-\beta )}=\sum_{C^{\left(1\right)}\in P_{1}^{\left(1\right)}}\;e^{-E_{C^{\left(1\right)}}}\cdot g_{1}\cdot e^{\varepsilon (n_{1},n_{1}-\beta )},$$

where $g_{1}=\sum_{C^{\left(2\right)}\in P_{1}^{\left(2\right)}}\;e^{-E_{C^{\left(2\right)}}}$. *g*_1_ represents the sum of Boltzmann factor over all path starting from (*n*_1_,*n*_1_ − *β*) and ending at (*i,j*). According to previous statement that *n** < *i*,*j* < *n** + *n*_1_−*β*, we have *I* − *n*_1_ < *n** − *β* < *n** and *j* − (*n*_1_ − *β*) < *n*.*This implies that the difference between coordinates of corresponding starting point (*n*_1_,*n*_1_ − *β*) and ending point (*i,j*), i.e., *I* − *n*_1_ and *j* − *n*_1_+*β*, is not greater than *n** respectively. So, *g*_1_ is computable because not greater *n** pair data are used. Obviously, the first term on the most right hand side (RHS) of Equation (A3) is computable also, which resulted from similar analysis with *g*_1_. In a word, the cumulative Boltzman factor shown as the term on the most left hand side (LHS) of (A3) is computable.

② When *C*∈P_2_ corresponding the case of *K* = 1, we have:

*C* = *C*^(1)^ + *C*^(2)^ = *C*_(0,0)__→(*n*1, *n*1-_*_β+_*_1)_+ *C*_(*n*1, *n*1-_*_β+_*_1)__→(*i*,*j*)_ = *C*^(1)^ + *C*^(2)^ + (*n*_1_,*n*_1_ − *β*) − (*n*_1_,*n*_1_ − *β*).

In the expression above, let $C_{*}^{\left(2\right)}$ = *C*^(2)^ + (*n*_1_,*n*_1_−*β*) = *C*_(*n*1, *n*1-_*_β+_*_1)__→(*i*,*j*)_ + (*n*_1_,*n*_1_−*β*). $C_{*}^{\left(2\right)}$ represents a path starting from (*n*_1_,*n*_1_ − *β*) and ending at (*i*,*j*). We define:

P2(2)(a)=P2(2)+(n1,n1−β)={C*(2):C(2)+(n1,n1−β),C(2)∈P2(2)}.

The energy of path *C*∈P_2_ can be described by:

$$E_{C}=E_{C^{\left(1\right)}}+E_{C^{\left(2\right)}}-\varepsilon (n_{1},n_{1}-\beta +1)=E_{C^{\left(1\right)}}+E_{C_{*}^{\left(2\right)}}-\varepsilon (n_{1},n_{1}-\beta )-\varepsilon (n_{1},n_{1}-\beta +1).$$

Therefore, we have:

(A4) $$e^{-E_{C}}=e^{-E_{C^{\left(1\right)}}}\cdot e^{-E_{C_{*}^{\left(2\right)}}}\cdot e^{\left[\varepsilon (n_{1},n_{1}-\beta )+\varepsilon (n_{1},n_{1}-\beta +1)\right]},$$

(A5) ∑C∈P2e−EC=∑C(1)∈P2(1) e−EC(1)⋅∑C*(2)∈P2(2)(a) e−EC*(2)⋅e[ε(n1,n1−β)+ε(n1,n1−β+1)]=∑C(1)∈P2(1) e−EC(1)⋅g2⋅e[ε(n1,n1−β)+ε(n1,n1−β+1)].

③ When *C*∈P *_K_*_+1_ corresponding the general case, similarly we have:

$$\begin{array}{cc} E_{C} & =E_{C^{\left(1\right)}}+E_{C^{\left(2\right)}}-\varepsilon (n_{1},n_{1}-\beta +K)\\ & =E_{C^{\left(1\right)}}+E_{C_{*}^{\left(2\right)}}-\sum_{d=0}^{K-1}\varepsilon (n_{1},n_{1}-\beta +d)-\varepsilon (n_{1},n_{1}-\beta +K)\\ & =E_{C^{\left(1\right)}}+E_{C_{*}^{\left(2\right)}}-\sum_{d=0}^{K}\varepsilon (n_{1},n_{1}-\beta +d) \end{array}$$

Furthermore, we have:

(A6) ∑C∈Pk+1e−EC=∑C(1)∈Pk+1(1) e−EC(1)⋅∑C*(2)∈Pk+1(2)(a) e−EC*(2)⋅e∑d=0kε(n1,n1−β+d),

where path $C_{*}^{\left(2\right)}$ and path set Pk+1(2)(a) are defined as follows:

C*(2)=C(2)+(n1,n1−β+K−1)+⋯+(n1,n1−β)Pk+1(2)(a)=Pk+1(2)+(n1,n1−β+K−1)+⋯+(n1,n1−β)={C*(2):(n1,n1−β)→(n1,n1−β+1)→⋯→(n1,n1−β+K)→(i,j)}

More specifically, in this case, *C*^(2)^ represents a path from (*n*_1_,*n*_1_ − *β* + *K*) to point (*i,j*); and $C_{*}^{\left(2\right)}$ represents a path starting from (*n*_1_,*n*_1_ − *β*) and passing through points (*n*_1_,*n*_1_ − *β* + 1), (*n*_1_,*n*_1_ – *β* + 2),…, (*n*_1_,*n*_1_ − *β* + *K −*1), (*n*_1_,*n*_1_ – *β* + *K*) and ending at point (*i,j*). Pk+1(2)(a) is a path set including all paths like $C_{*}^{\left(2\right)}$. Furthermore, we define:

Pk+1(1)(a)=Pk+1(1)+(n1,n1−β+K+1)+⋯+(n1,n1+β)={C*(1):(0,0)→(n1,n1−β+K)→(n1,n1−β+K+1)→⋯→(n1,n1+β)}.

$C_{*}^{\left(1\right)}$ in the definition above represents the path starting from (0,0) and arriving at point (*n*_1_,*n*_1_-*β*+*K*) first and then passing through (*n*_1_,*n*_1_ – *β* + *K* + 1), (*n*_1_,*n*_1_ – *β* + *K* + 2),…, (*n*_1_,*n*_1_ + *β* − 1) and ending at (*n*_1_,*n*_1_ + *β*). Moreover, $C_{*}^{\left(1\right)}$ does not include anyone among the points (*n*_1_,*n*_1_ − *β*), (*n*_1_,*n*_1_ − *β +* 1),…, (*n*_1_,*n*_1_ − *β* + *K* − 1). According to the definition of $C_{*}^{\left(1\right)}$, we have:

(A7) $$E_{C_{*}^{\left(1\right)}}=E_{C^{\left(1\right)}}+\varepsilon (n_{1},n_{1}-\beta +K+1)+\cdots +\varepsilon (n_{1},n_{1}+\beta )=E_{C^{\left(1\right)}}+\sum_{d=K+1}^{2\beta }\varepsilon (n_{1},n_{1}-\beta +d).$$

Equivalently, we have

(A8) $$E_{C^{\left(1\right)}}=E_{C_{*}^{\left(1\right)}}-\sum_{d=K+1}^{2\beta }\varepsilon (n_{1},n_{1}-\beta +d).$$

Substituting (A8) into the first term of right hand side of Equation (A6), we have:

(A9) ∑C∈Pk+1e−EC=∑C*(1)∈Pk+1(1)(a) e−EC*(1)⋅∑C*(2)∈Pk+1(2)(a) e−EC*(2)⋅exp{∑K=02βε(n1,n1−β+K)}.

According to the definition of PK+1(2)(a), we have P1(2)=P1(2)(a)⊃P2(2)(a)⊃⋯⊃P2β+1(2)(a). Let *g_K_*_+1_ denote the second term of RHS of Equation (A9). *g_K_*_+1_ should decrease with *K* increasing, i.e., *g*_2_*_β_*_+1_ ≤ *g*_2_*_β_* ≤ …≤ *g_K_*_+1_ ≤ … ≤ *g*_2_ ≤ *g*_1_. More specifically, *g*_1_ is the maximum value among {*g_K_*_+1_, *K* = 0,1,…,2*β*} and represents the cumulative Boltzman factor corresponding to all path from (*n*_1_,*n*_1_ − *β*) to (*i*,*j*) under the condition |*j* − *i*| ≤ *β* and *n** < *i*,*j* < *n** + *n*_1_ − *β*. According to the analysis of case ①, *g*_1_ is computable. *g*_2_*_β_*_+1_ is the minimum value {*g_K_*_+1_, *K* = 0,1,…,2*β*} and represents the multiplication of the Boltzman factor corresponding to the path from (*n*_1_,*n*_1_ − *β*) to (*n*_1_,*n*_1_ + *β* − 1) and the cumulative Boltzman factor corresponding to all path from (*n*_1_,*n*_1_ + *β*) to (*i*,*j*). *g*_2_*_β_*_+1_ is computable also, because *g*_2_*_β_*_+1_ is less than computable *g*_1_. We use the average value of *g*_1_ and *g*_2_*_β_*_+1_, denoted by *r_i_*_,*j*_ to approximate the values of all *g_K_*_+1_ (*K* = 0,1,…,2*β*) for the convenience of following computations and analyses. The third term of right hand side of Equation (A9) is a constant value and can be denoted by *D*. We substitute *r_i_*_,*j*_ and *D* to the second and third terms of right hand side of (A9) respectively and we have:

(A10) ∑C∈Pk+1e−EC≈∑C*(1)∈Pk+1(1)(a) e−EC*(1)⋅ri,j⋅D.

Furthermore, we can obtain the cumulative Boltzman factor corresponding to all path from (0,0) to (*i*,*j*) by the following expression:

(A11) g(i,j)=∑Ce−EC=∑K=02β∑C∈PK+1e−EC≈∑K=02β∑C*(1)∈Pk+1(1)(a) e−EC*(1)⋅ri,j⋅D=g(n1,n1+β)⋅ri,j⋅D .

In (A11), *g*(*n*_1_,*n*_1_ + *β*) denotes the cumulative Boltzman factor corresponding all possible paths from (0,0) to (*n*_1_,*n*_1_ + *β*). According to the previous definition of PK+1(1)(a), ∪K=02βPK+1(1)(a) is the path set including all paths starting from (0,0) and ending at (*n*_1_,*n*_1+_*_β_*) and PK+1(1)(a) are disjoint sets for different *K*. Therefore, the sum of the cumulative Boltzman factor corresponding to each path set PK+1(1)(a) is exactly the cumulative Boltzman factor corresponding all possible paths from (0,0) to (*n*_1_,*n*_1_ + *β*) and can surely be denoted by *g*(*n*_1_,*n*_1_ + *β*). So far, we have approximately expressed the cumulative Boltzman factor from (0,0) to (*i*,*j*), *g*(*i*,*j*) by the multiplication of the cumulative Boltzman factor from (0,0) to(*n*_1_,*n*_1_ + *β*), *g*(*n*_1_,*n*_1_ + *β*) and the average value of the cumulative Boltzman factor from (*n*_1_,*n*_1_ + *β*) to (*i*,*j*) and the cumulative Boltzman factor from (*n*_1_,*n*_1_ − *β*) to(*i*,*j*), *r_i_*_,*j*_, when *n** < *i*, *j* < *n** + *n*_1_ − *β*. Furthermore, we can compute the average thermal path as follows:

(A12) $$<x(t)>=\sum_{i=\max\{0,t-n,\frac{t-\beta -\delta }{2}\}}^{\min\{n,t,\frac{t+\beta +\delta }{2}\}}\;\;(t-2i)g(i,t-i)/\sum_{i=\max\{0,t-n,\frac{t-\beta -\delta }{2}\}}^{\min\{n,t,\frac{t+\beta +\delta }{2}\}}\;\;g(i,t-i)\;\approx \sum_{i=\max\{0,t-n,\frac{t-\beta -\delta }{2}\}}^{\min\{n,t,\frac{t+\beta +\delta }{2}\}}\;\;(t-2i)\cdot g(n_{1},n_{1}+\beta )\cdot r_{i,t-i}\cdot D/\sum_{i=\max\{0,t-n,\frac{t-\beta -\delta }{2}\}}^{\min\{n,t,\frac{t+\beta +\delta }{2}\}}\;\;g(n_{1},n_{1}+\beta )\cdot r_{i,t-i}\cdot D\;=\sum_{i=\max\{0,t-n,\frac{t-\beta -\delta }{2}\}}^{\min\{n,t,\frac{t+\beta +\delta }{2}\}}\;\;(t-2i)\cdot r_{i,t-i}/\sum_{i=\max\{0,t-n,\frac{t-\beta -\delta }{2}\}}^{\min\{n,t,\frac{t+\beta +\delta }{2}\}}r_{i,t-i}.$$

In order to obtain the expression of 〈x(t)〉 above and analyze conveniently, we suppose that all *i* and *t* − *i* in (A12) are between *n** and *n** + *n*_1_ − *β*. In fact, only if the maximum value of independent variable *i* of *g*(*i*,*t* − *i*) used in (15) to compute 〈x(t)〉 is between *n** and *n** + *n*_1_ − *β*, the thermal average path 〈x(t)〉 can be obtained by (A12). Let us give more detailed explanation. If the maximum value of *i* in all *g*(*i*,*t* − *i*) of expression (15) is greater than *n**, then the minimum value of *i* must be greater than *n** − *β*, due to condition of Pruning Algorithm shown by (14). This implies that all possible values of independent variables pairs *i* and *t* − *i* of *g*(*i*,*t* − *i*) involved in expression (15) are greater than *n** − *β*. It is clear that we have all *i* and *t* − *i* are greater than *n** − *β* also greater than *n*_1_ + *β*, since we suppose *n** ≥ *n*_1_ + 3*β*. Thus, *g*(*i*,*t* − *i*) in that all *i* and *t* − *i* are greater than *n*_1_ + *β* can approximately be described by (A11) and 〈x(t)〉 can be determined by expression (A12) correspondingly, based on the previous analyses. It tells that only if exist one term *g*(*i*,*t* − *i*) used in (15) to compute 〈x(t)〉 is unacceptably large, all other related terms *g*(*i*,*t* − *i*) should take quite big value, therefore all used *g*(*i*,*t* − *i*) can be described by (A11) and therefore, 〈x(t)〉 can be determined by expression (A12).

Now, we can summarize that expression (A12), called Path segmentation algorithm in this paper, can be used to obtain 〈x(t)〉, when the maximum value of independent variable *i* of *g*(*i*,*t* − *i*) used in (15) to compute 〈x(t)〉 is between *n** and *n** + *n*_1_ − *β*.

Let us discuss the case when the maximum value of independent variable *i* of *g*(*i*,*t* − *i*) used in (15) to compute 〈x(t)〉 is greater than *n** + *n*_1_ − *β* and less than *n*. We change the boundary of segmentation path from {(*t*_1_,*t*_2_): *t*_1_ = *n*_1_, *n*_1_ – *β* ≤ *t*_2_ ≤ *n*_1_ + *β*, *t*_1_ and *t*_2_ are integers} to {(*t*_1_,*t*_2_): *t*_1_ = 2*n*_1_, 2*n*_1_ – *β* ≤ *t*_2_ ≤ 2*n*_1_ + *β*, *t*_1_ and *t*_2_ are integers}. This is clear that the computation of *g*(*i*,*t* − *i*) with such *i* that is greater than *n** + *n*_1_ − *β*may involve *n** or more than *n** pair data, which is likely to result in unacceptably large value of *g*(*i*,*t* − *i*), if we choose {(*t*_1_,*t*_2_): *t*_1_ = *n*_1_, *n*_1_ − *β* ≤ *t*_2_ ≤ *n*_1_ + *β*, *t*_1_ and *t*_2_ are integers} as the boundary of segmentation path, since *i*−(*n*_1_ − *β*) > *n** + *n*_1_ − *β* − (*n*_1_ − *β*) = *n**. In this case, the minimum value of independent variable *i* of *g*(*i*,*t* − *i*) used in (15) to compute 〈x(t)〉 should be greater than (*n** + *n*_1_ − *β*) − *β = n** + *n*_1_ − 2*β*, since the difference between the maximum value and minimum value of independent variable *i* of *g*(*i*,*t* − *i*) is not greater than *β*. Furthermore, we have that all possible values of the two independent variables *i* and *t* − *i* of *g*(*i*,*t* − *i*) used in (15) to compute 〈x(t)〉 are between *n** + *n*_1_ − 2*β* and *n*. Similarly to the case when *n** < *i*, *j =* *t* – *I* < *n** + *n*_1_ − *β*, all path starting from (0,0) and ending to (*i*,*t* − *i*) in this case can be divided into two parts, the one from (0,0) to (2*n*_1_,2*n*_1_ − *β* + *K*) and the another one from (2*n*_1_,2*n*_1_ – *β* + *K*) to (*i*,*t* − *i*) where *K* = 0,1,…,2*β*, since *i* − (2*n*_1_ *+* *β*) > *n** + *n*_1_ − 2*β*−(2*n*_1_*+**β*) = *n** − (*n*_1_ + 3*β*)>0, *t* − *i* − (2*n*_1_ + *β*) > *n** − (*n*_1_ + 3*β*) > 0. Correspondingly, we have:

(A13) $$g(i,j)=\sum_{C}e^{-E_{C}}\approx g(2n_{1},2n_{1}+\beta )\cdot r_{i,j}\cdot D\;,$$

where *r_i_*_,*j*_ is the average value of the cumulative Boltzman factor from (2*n*_1_,2*n*_1_ + *β*) to (*i*,*j*) and the cumulative Boltzman factor from (2*n*_1_,2*n*_1_ − *β*) to (*i*,*j*); and *D* is a constant equal to $exp\{\sum_{K=0}^{2\beta }\varepsilon (2n_{1},2n_{1}-\beta +K)$. Furthermore, we have:

(A14) $$\begin{array}{l} <x(t)>=\sum_{i=\max\{0,t-n,\frac{t-\beta -\delta }{2}\}}^{\min\{n,t,\frac{t+\beta +\delta }{2}\}}\;\;(t-2i)g(i,t-i)/\sum_{i=\max\{0,t-n,\frac{t-\beta -\delta }{2}\}}^{\min\{n,t,\frac{t+\beta +\delta }{2}\}}\;\;g(i,t-i)\\ \approx \sum_{i=\max\{0,t-n,\frac{t-\beta -\delta }{2}\}}^{\min\{n,t,\frac{t+\beta +\delta }{2}\}}\;\;(t-2i)\cdot g(2n_{1},2n_{1}+\beta )\cdot r_{i,t-i}\cdot D/\sum_{i=\max\{0,t-n,\frac{t-\beta -\delta }{2}\}}^{\min\{n,t,\frac{t+\beta +\delta }{2}\}}\;\;g(2n_{1},2n_{1}+\beta )\cdot r_{i,t-i}\cdot D\\ =\sum_{i=\max\{0,t-n,\frac{t-\beta -\delta }{2}\}}^{\min\{n,t,\frac{t+\beta +\delta }{2}\}}\;\;(t-2i)\cdot r_{i,t-i}/\sum_{i=\max\{0,t-n,\frac{t-\beta -\delta }{2}\}}^{\min\{n,t,\frac{t+\beta +\delta }{2}\}}r_{i,t-i}.\; \end{array}$$

Finally, we can summarize that expression (A14), also called Path segmentation algorithm in this paper, can be used to obtain 〈x(t)〉, when the maximum value of independent variable *i* of *g*(*i*,*t* − *i*) used in (15) to compute 〈x(t)〉 is between *n** + *n*_1_ – *β* and *n*. The appendix is an optional section that can contain details and data supplemental to the main text. For example, explanations of experimental details that would disrupt the flow of the main text, but nonetheless remain crucial to understanding and reproducing the research shown; figures of replicates for experiments of which representative data is shown in the main text can be added here if brief, or as Supplementary data. Mathematical proofs of results not central to the paper can be added as an appendix.
